# Editorial Department

**Published:** 1880-01

**Authors:** 


### EDITORIAL DEPARTMENT.

### THE OBJECTS AND SCOPE OF THE ARCHIVES.

We present herewith to the Veterinary Profession of America a Quarterly Journal devoted to Comparative Medical Science.

In offering this Journal to the profession, we have at least this excuse, that such a one is as yet a desideratum. Whether our present undertaking answers all the requirements of the situation or no, the reader will doubtless decide for himself. Our aim is to make it as valuable in the department of original contributions, and as comprehensive in its summary of what has been and is done abroad, as our best efforts can render it.

The Archives will contain the following Departments :

I. Original Articles, Selections, and Translations.—We have secured the services of the most eminent Veterinary Teachers, practical Veterinarians, and Physicians interested in Comparative Medicine in the United States, Canada, and Germany, for this department.

II. Editorial Department.—Herein topies of special interest to the Veterinarian, such as those involved in Veterinary Legislation, higher Veterinary Education, etc., will be discussed by the Editor and his Staff.

III. Abstract and Summary of Comparative Medical Science.—This department will contain abstracts of all important treatises on subjects related to Veterinary Medicine from the English, German, French, and domestic literature. It is proposed to make this one of the most valuable departments of the "Archives."

IV. The Review Department will consist of reviews of the more important veterinary text-books and monographs appearing in each quarter of the year.

V. MISCELLANEOUS DEPARTMENT.—This will contain Reports of the more important and interesting autopsies, dissections, and cases observed during the quarter, also any other items of general interest as may merit the attention of the profession. Several reports of operations are deferred to the next number of the "Archives," considerable space being occupied in the present issue by the introductory lecture delivered at one of our Veterinary Schools.

While in one sense we place the "Archives" in competition with similar periodicals appearing on the other side of the Atlantic, we, in another sense, will cheerfully confess that, for the first few years at least, our aim cannot be as high.

The neglected condition of veterinary education in this country renders it incumbent on any journal addressed to the American veterinary profession to adopt a simpler tone than its transatlantic compeers. It must be more *didactic*, in the ordinary sense of that word, than the latter; deal with simpler issues, and be addressed to the student as well as the ripe practitioner. Hence the combination in the "Archives" of articles which, we trust, can be ranked as elaborate monographs, with others which are more comparable to lectures directed to a veterinary class. However much this association may appear anomalous to transatlantic veterinarians, we can assure them that the circumstances fully warrant it. Until Veterinary Science has reached the same level here as in Europe, and perhaps also in Canada, our "Archives" shall not venture to simulate in every respect the excellent journals of the Old World.

The interest which members of the human medical profession have taken and are taking in comparative medical science, as well as the encouragement which our undertaking has received at their hands, encourages us to hope for a continuance and an extension of these favors, from those whose field of study and research that of the Veterinarian is so closely kin to.

### "A NATIONAL BUREAU OF VETERINARY INSPECTION."

The New York *Medical Record*, under the above heading, presents an item which is of considerable interest to the Veterinarian, and which we have therefore inserted in full on one of the last pages of the "Archives." The propositions in it merit our full endorsement. In the first place, the American Veterinary Association is not a body analogous to our National Medical Association; it is not composed of such elements as can claim the reverence due to scientific authority, and its *dicta* may be readily disposed of as possessing but a slight, if any intrinsic value. What the writer of that item says in reference to the fact that such a board would have no more legitimate or professional basis than a National Bureau of Barbers, is strictly correct, especially if we picture to ourselves this Board as it would be, presumably selected from among certain of the members of the American Veterinary Association.

The entire proposal savoreth strongly of what is technically known as a "job." All the work laid out for such a "Board" can be accomplished far more thoroughly by our already existing medical boards, and we can see no reason why the whole matter might not be safely confided to the Army Medical Department.

There is one single error in the writer's assumptions, which is merely a collateral one, but we feel compelled to refer to it. There are altogether three veterinary schools in this country, all of them in New York City, and all authorized to grant regular diplomas—not *one*, as stated by him. Of these three schools the one possessing the oldest charter consists of a building now used as a *hospital stable* by a veterinary surgeon; no lectures are given, nor any students visible around the deserted premises of its lecture-room. This is the New York College of Veterinary Surgeons. Two years ago the whole Faculty of this school, with the exception of two members, one of whom was accused of incapacity, (the other being neither a physician nor a veterinarian,) left this institution in a body and organized a new school, the "Columbia Veterinary College." *All* the students of the New

York College, numbering eighteen, disgusted with the action of the Board of Trustees of the New York College, one of whose members had utilized the name of the college in advertising his private nostrums, followed their faculty to the new college. The latter is the *only* veterinary college in America in which the branches of Comparative Pathology, Pharmacy, Therapeuties, Physiology, Chemistry, Breeding of domestic animals, and cattle practice are taught on the same basis as the analogous branches are taught in our better medical schools and the veterinary schools of Germany and England. It is, we believe, the only school on this side of the Atlantic in which there is a special chair for the branch of veterinary jurisprudence, and that most important branch, "practical shoeing as applied to the normal and pathological foot." There is one other regular veterinary school, which, if we are not misinformed, was organized by gentlemen who had left the "New York College" on account of some similar dissensions with its Board of Trustees.

In *organization* there is only one fundamental difference between the two veterinary schools, (that is, the two that have an actual existence). The Columbia Veterinary College does *not* offer free scholarships to agricultural or any other societies. Its Faculty and Trustees hold that if agricultural societies take sufficient interest in veterinary medicine to send young men to the city for purposes of studying that science, that they are fully able and should be held to pay for the instruction given. The same Faculty and Trustees are, as we are informed, also of the opinion that the reception of students free of any other charges save the "graduation fee" is apt to conflict with the true interests of veterinary teaching, by acting as a *quasi* temptation to graduating incompetent men for the mere sake of increasing the receipts of the institution.

The correspondent of the *Medical Record*, if acquainted with the true state of things, would not have referred to the two last schools mentioned as merely empowered to grant certificates. The diploma of either is at least fully as "regular" as that granted by some of our acknowledged medical schools, while the only especially chartered college, to which the writer seems to refer, is without a single student, and has been placed in a rather ambiguous position by its Trustees, so that reputable medical men and veterinarians have declined to be associated with it.

The best qualifications of the veterinary graduate are not derived from the date of any given college charter, but from the character of the curriculum and the thoroughness of the examination through which he has passed.

To return from this digression, we would state that there is no cause for apprehending that the scheme of the American Veterinary Association has the remotest chance of becoming realized; we refer to it only in order to give the profession an idea of the projects which a certain circle would carry out if they had the power.

